# Supplementary material for: CPEB1 directs muscle stem cell activation by reprogramming the translational landscape
Source: Nat Commun. 2022 Feb 17;13:947. doi: 10.1038/s41467-022-28612-1 (PMC8854658; doi:10.1038/s41467-022-28612-1)
Supplement: Supplementary file 1 — Supplementary Information [file 41467_2022_28612_MOESM1_ESM.pdf]

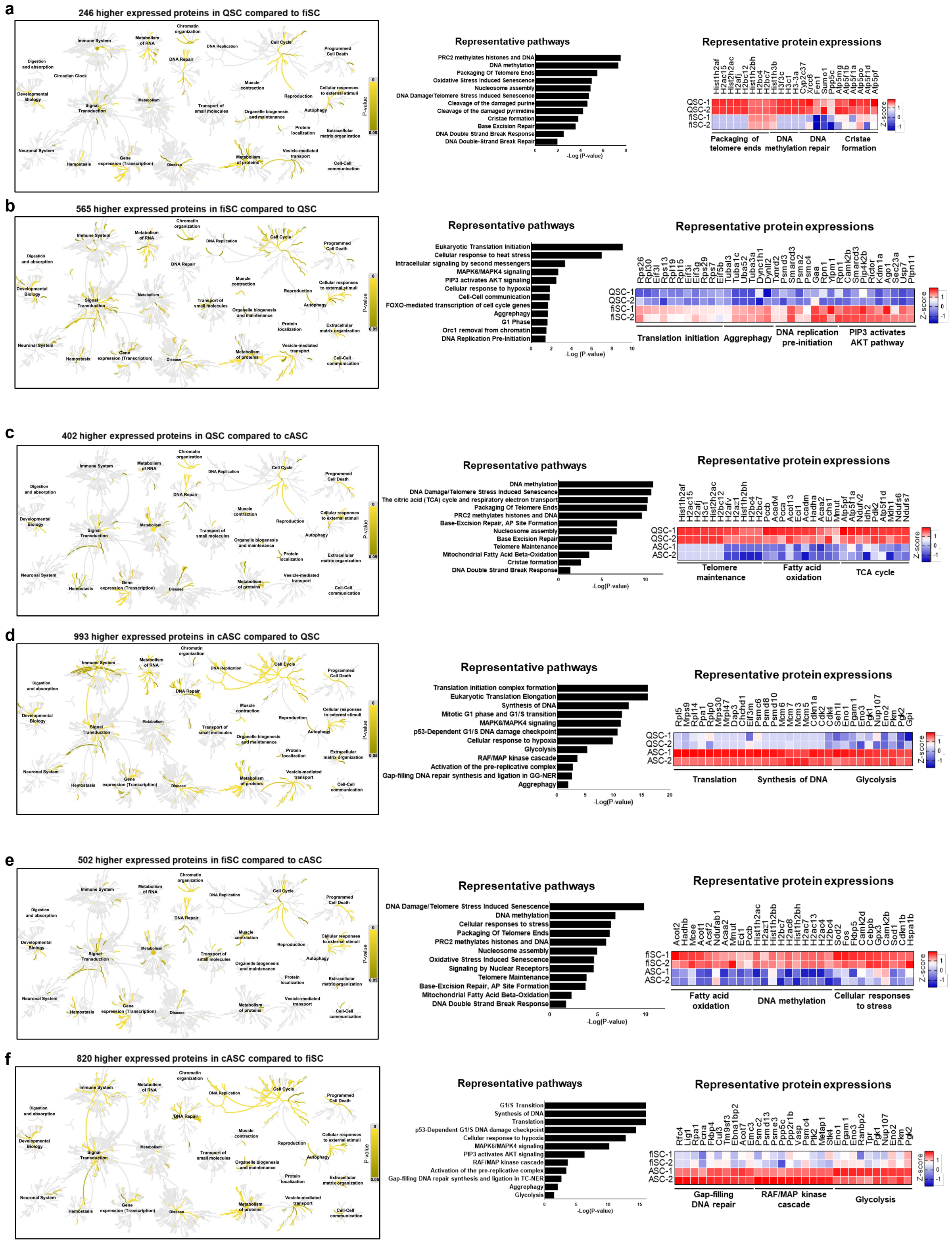

a
246 higher expressed proteins in QSC compared to fiSC
Representative pathways
PRC2 methylates histones and DNA
DNA methylation
Packaging Of Telomere Ends
Oxidative Stress Induced Senescence
Nucleosome assembly
DNA Damage/Telomere Stress Induced Senescence
Cleavage of the damaged purine
Cleavage of the damaged pyrimidine
Cristae formation
Base Excision Repair
DNA Double Strand Break Response
DNA Double-Strand Break Repair
-Log (P-value)
Representative protein expressions
Packaging of telomere ends
DNA methylation
DNA repair
Cristae formation
Z-score
b
565 higher expressed proteins in fiSC compared to QSC
Representative pathways
Eukaryotic Translation Initiation
Cellular response to heat stress
Intracellular signaling by second messengers
MAPK6/MAPK4 signaling
PIP3 activates AKT signaling
Cellular response to hypoxia
Cell-Cell communication
FOXO-mediated transcription of cell cycle genes
Aggrephagy
G1 Phase
Orc1 removal from chromatin
DNA Replication Pre-Initiation
-Log (P-value)
Representative protein expressions
Translation initiation
Aggrephagy
DNA replication pre-initiation
PIP3 activates AKT pathway
Z-score
c
402 higher expressed proteins in QSC compared to cASC
Representative pathways
DNA methylation
DNA Damage/Telomere Stress Induced Senescence
The citric acid (TCA) cycle and respiratory electron transport
Packaging Of Telomere Ends
PRC2 methylates histones and DNA
Base-Excision Repair, AP Site Formation
Nucleosome assembly
Base Excision Repair
Telomere Maintenance
Mitochondrial Fatty Acid Beta-Oxidation
Cristae formation
DNA Double Strand Break Response
-Log(P-value)
Representative protein expressions
Telomere maintenance
Fatty acid oxidation
TCA cycle
Z-score
d
993 higher expressed proteins in cASC compared to QSC
Representative pathways
Translation initiation complex formation
Eukaryotic Translation Elongation
Synthesis of DNA
Mitotic G1 phase and G1/S transition
MAPK6/MAPK4 signaling
p53-Dependent G1/S DNA damage checkpoint
Cellular response to hypoxia
Glycolysis
RAF/MAP kinase cascade
Activation of the pre-replicative complex
Gap-filling DNA repair synthesis and ligation in GG-NER
Aggrephagy
-Log(P-value)
Representative protein expressions
Translation
Synthesis of DNA
Glycolysis
Z-score
e
502 higher expressed proteins in fiSC compared to cASC
Representative pathways
DNA Damage/Telomere Stress Induced Senescence
DNA methylation
Cellular responses to stress
Packaging Of Telomere Ends
PRC2 methylates histones and DNA
Nucleosome assembly
Oxidative Stress Induced Senescence
Signaling by Nuclear Receptors
Telomere Maintenance
Base-Excision Repair, AP Site Formation
Mitochondrial Fatty Acid Beta-Oxidation
DNA Double Strand Break Response
-Log(P-value)
Representative protein expressions
Fatty acid oxidation
DNA methylation
Cellular responses to stress
Z-score
f
820 higher expressed proteins in cASC compared to fiSC
Representative pathways
G1/S Transition
Synthesis of DNA
Translation
p53-Dependent G1/S DNA damage checkpoint
Cellular response to hypoxia
MAPK6/MAPK4 signaling
PIP3 activates AKT signaling
RAF/MAP kinase cascade
Activation of the pre-replicative complex
Gap-filling DNA repair synthesis and ligation in TC-NER
Aggrephagy
Glycolysis
-Log(P-value)
Representative protein expressions
Gap-filling DNA repair
RAF/MAP kinase cascade
Glycolysis
Z-score

Supplementary Fig. 1 (associated to Fig. 1). Significant changes in the SC proteome during the quiescence-to-activation transition

(a, b) Proteome comparison of QSCs to fiSCs. (a) A global view of Reactome functional enrichment analysis of 246 proteins highly expressed in QSCs when compared to fiSCs. The bar chart shows the representative pathways that the 246 proteins in QSCs are enriched. Heatmap shows the representative highly expressed proteins in QSCs. (b) A global view of Reactome functional enrichment analysis of 565 upregulated proteins in fiSCs. Bar chart showed the representative pathways that upregulated proteins in fiSCs are enriched. Heatmap showed the representative highly expressed proteins in fiSCs.

(c, d) Proteome comparison of QSCs to cASCs. (c) A global view of Reactome functional enrichment analysis of 402 proteins highly expressed in QSCs when compared to cASCs. The bar chart shows the representative pathways that the 402 proteins in QSCs are enriched. Heatmap shows the representative highly expressed proteins in QSCs. (d) A global view of Reactome functional enrichment analysis of 993 upregulated proteins in cASCs. Bar chart shows the representative pathways that upregulated proteins in cASCs are enriched. Heatmap shows the representative upregulated proteins in cASCs.

(e, f) Proteome comparison of fiSCs to cASCs. (e) A global view of Reactome functional enrichment analysis of 502 highly expressed proteins in fiSCs when compared to cASCs. The bar chart shows the representative pathways that the 502 proteins in fiSCs are enriched. Heatmap shows representative highly expressed proteins in fiSCs. (f) A global view of Reactome functional enrichment analysis of 820 upregulated proteins in cASCs. Bar chart shows the representative pathways that upregulated proteins in cASCs are enriched. Heatmap shows representative upregulated proteins in cASCs.

In a-f, the functional enrichment analysis was performed by Reactome. In Reactome, the method used to calculate statistical significance was the binomial test.

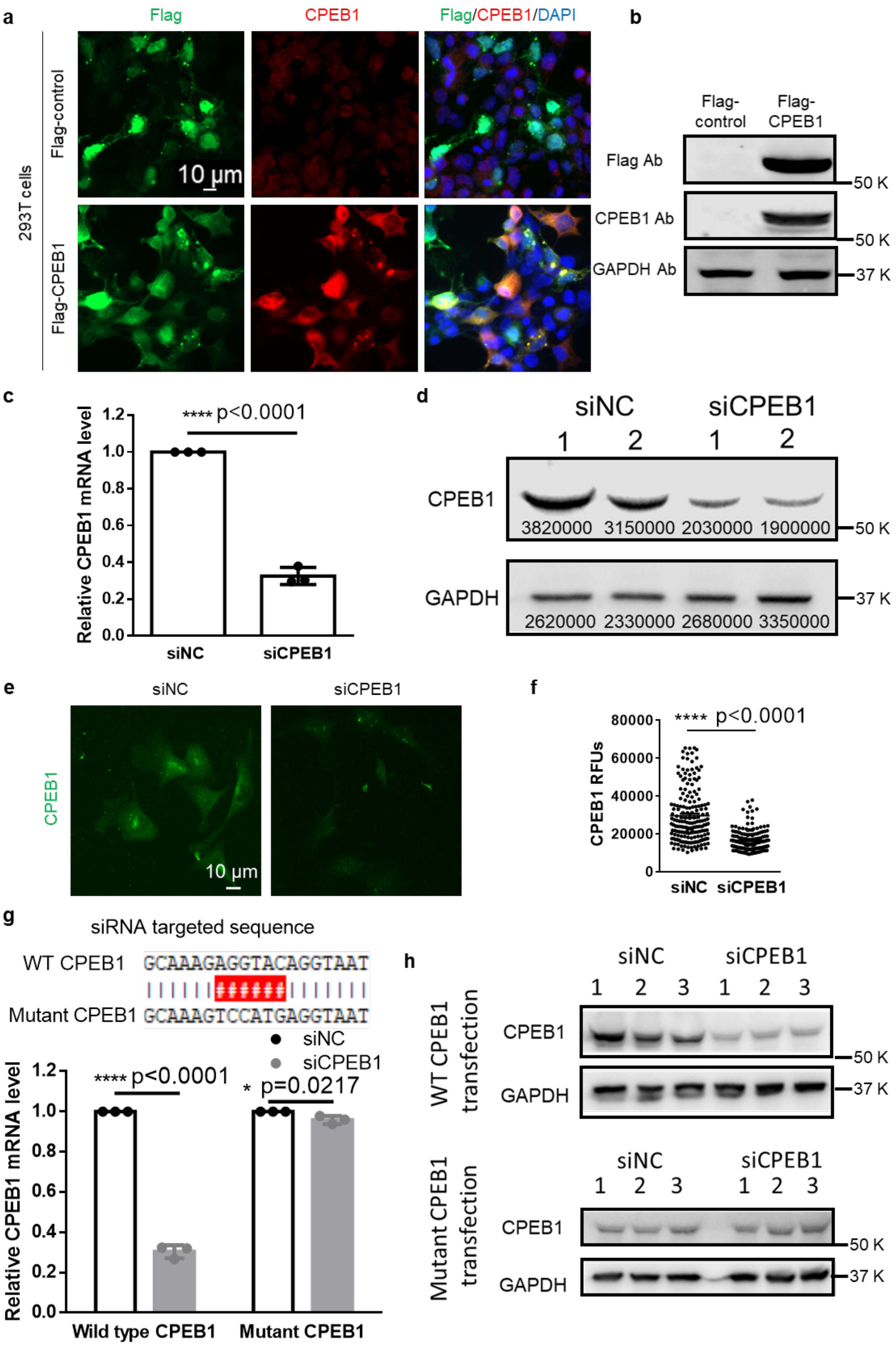
a
Flag
CPEB1
Flag/CPEB1/DAPI
293T cells
Flag-control
Flag-CPEB1
10 µm
b
Flag-control
Flag-CPEB1
Flag Ab
CPEB1 Ab
GAPDH Ab
50 K
50 K
37 K
c
**** p<0.0001
Relative CPEB1 mRNA level
1.2
1.0
0.8
0.6
0.4
0.2
0.0
siNC
siCPEB1
d
siNC
siCPEB1
1 2 1 2
CPEB1
3820000 3150000 2030000 1900000
50 K
GAPDH
2620000 2330000 2680000 3350000
37 K
e
siNC
siCPEB1
CPEB1
10 µm
f
**** p<0.0001
CPEB1 RFUs
80000
60000
40000
20000
0
siNC
siCPEB1
g
siRNA targeted sequence
WT CPEB1 GCAAAGAGGTACAGGTAAT
Mutant CPEB1 GCAAAGTCCATGAGGTAAT
siNC
siCPEB1
**** p<0.0001
* p=0.0217
Relative CPEB1 mRNA level
1.2
1.0
0.8
0.6
0.4
0.2
0.0
Wild type CPEB1
Mutant CPEB1
h
WT CPEB1 transfection
siNC
siCPEB1
1 2 3 1 2 3
CPEB1
GAPDH
50 K
37 K
Mutant CPEB1 transfection
siNC
siCPEB1
1 2 3 1 2 3
CPEB1
GAPDH
50 K
37 K

Supplementary Fig. 2 (associated to Fig. 2). CPEB1 antibody, siRNA specificity and efficiency tests

(a, b) CPEB1 antibody specificity test on 293T cells after Flag-control or Flag-CPEB1 overexpression. Immunostaining (n = 3 independent experiments) (a) and western blot (b) of CPEB1 and Flag after transfection of Flag-control or Flag-CPEB1 vector into 293T cells. GAPDH protein used as internal control. (n=3 independent experiments).

(c, d) CPEB1 siRNA efficiency test performed on primary myoblasts. (c) CPEB1 mRNA real-time PCR experiments were performed 48 hours after indicated siRNA transfection. (n=3 independent experiments). (d) Western blot for CPEB1 protein performed 48 hours after siRNA transfection. (n=2 independent experiments). The protein band intensity is shown below the corresponding protein band. GAPDH protein used as internal control.

(e, f) CPEB1 siRNA efficiency test performed on sorted SCs. (e) CPEB1 protein immunostaining was performed 48 hours after siRNA transfection on fiSCs. (f) Quantification of CPEB1 RFUs of SCs in (e). (n=3 independent experiments, the number of quantified cells is 180 and 156 for siNC and siCPEB1 respectively).

(g, h) CPEB1 siRNA specificity test performed on 293T cells. WT or mutant CPEB1 plasmid was transfected into 293T cells for 24 hours followed by CPEB1 siRNA transfection for 48 hours. (g) CPEB1 mRNA real-time PCR experiments were performed. (n=3 independent experiments). (h) CPEB1 protein western blot. (n=3 independent experiments). GAPDH protein used as internal control.

Data are presented as mean ± SD in c, f, and g. The *p* values calculated by two-tailed unpaired t test were used for comparing two groups in c, f, g, ns not significant. Source data is provided as a Source Data file.

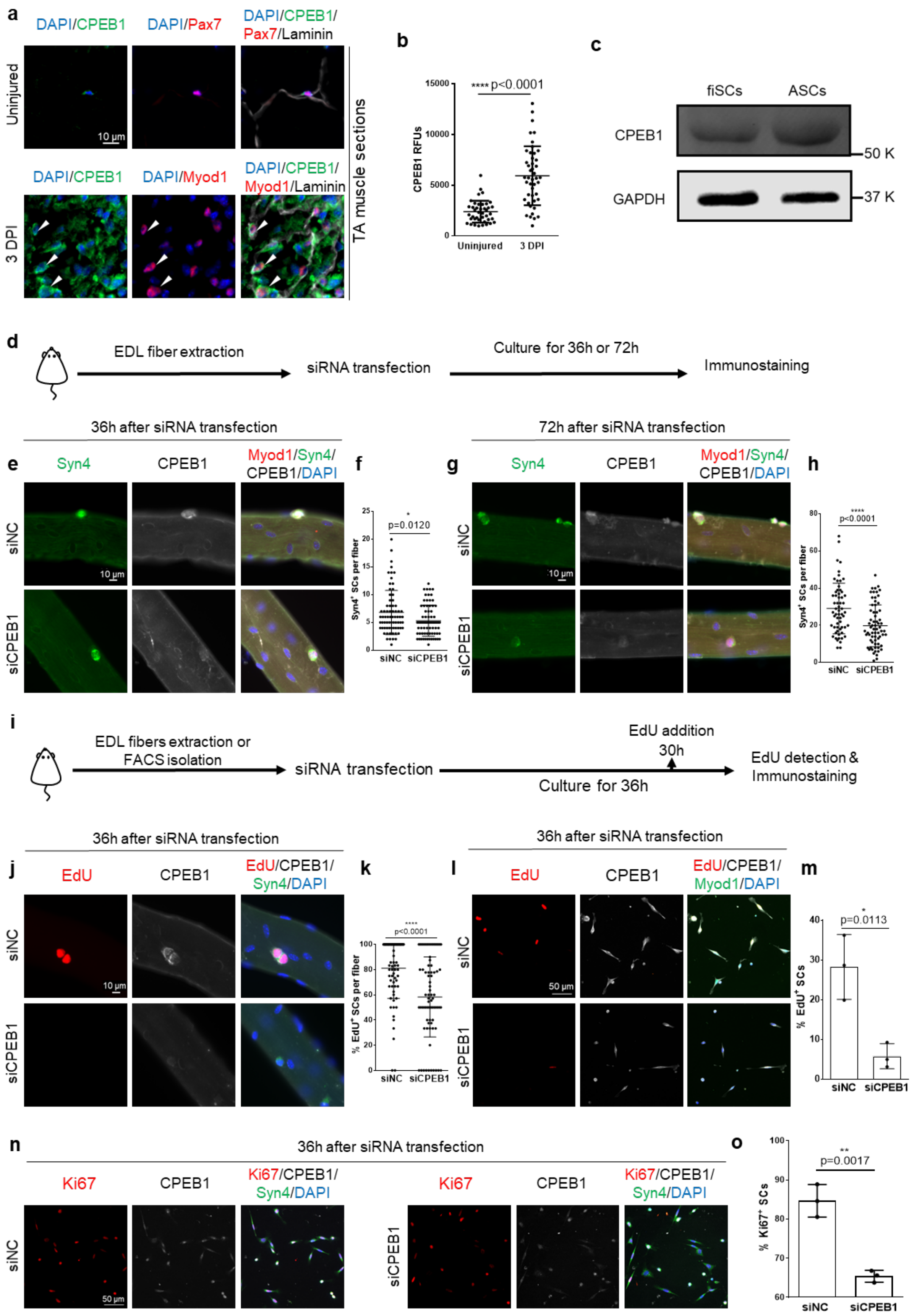

a
DAPI/CPEB1
DAPI/Pax7
DAPI/CPEB1/Pax7/Laminin
Uninjured
10 µm
DAPI/CPEB1
DAPI/Myod1
DAPI/CPEB1/Myod1/Laminin
3 DPI
TA muscle sections
b
**** p<0.0001
CPEB1 RFUs
Uninjured
3 DPI
c
fiSCs
ASCs
CPEB1
GAPDH
50 K
37 K
d
EDL fiber extraction
siRNA transfection
Culture for 36h or 72h
Immunostaining
36h after siRNA transfection
72h after siRNA transfection
e
Syn4
CPEB1
Myod1/Syn4/CPEB1/DAPI
siNC
siCPEB1
f
p=0.0120
Syn4+ SCs per fiber
g
h
p<0.0001
i
EDL fibers extraction or FACS isolation
siRNA transfection
EdU addition
30h
Culture for 36h
EdU detection & Immunostaining
j
EdU
EdU/CPEB1/Syn4/DAPI
k
p<0.0001
% EdU+ SCs per fiber
l
EdU/CPEB1/Myod1/DAPI
50 µm
m
p=0.0113
% EdU+ SCs
n
Ki67
Ki67/CPEB1/Syn4/DAPI
o
p=0.0017
% Ki67+ SCs

Supplementary Fig. 3 (associated to Fig. 2). CPEB1 is upregulated during activation and required for SC proliferation

(a - c) CPEB1 protein expression analysis on SCs of uninjured and 3 days post-injured (3 DPI) activated SCs. (a) CPEB1 immunostaining of QSCs (marked by Pax7 protein) from uninjured or ASCs (marked by Myod1 protein) of 3 DPI TA muscle sections. Laminin stains the muscle fibers. Nuclei were stained with DAPI. (b) Quantification of CPEB1 RFUs in SCs on TA muscle sections. (n=3 independent experiments, the number of cells for quantification is 41 and 45 for uninjured and 3 DPI muscle sections respectively). (c) Western blot of CPEB1 on fiSCs and ASCs (SCs sorted from 3 days post-injured muscles). GAPDH protein used as internal control. (n=3 independent experiments).

(d - h) Analysis of fiber-associated SCs number after CPEB1 knockdown. (d) Schematic illustration of SCs number analysis on CPEB1 knocked down fibers. (e, g) 36 or 72 hours after siRNA transfection, fibers were subjected to immunostaining for Syn4, Myod1, and CPEB1. (f, h) Quantification of $Syn4^{+}$ SCs number per fiber after siRNA transfection. In (f), n=3 independent experiments, the number of quantified fibers is 71 and 74 for siNC and siCPEB1 respectively. In (h), n=3 independent experiments, the number of quantified fibers is 60 and 67 for siNC and siCPEB1 respectively.

(i - m) EdU incorporation analysis in SCs after CPEB1 knockdown. (i) Schematic illustration of the EdU incorporation analysis on CPEB1 knocked down SCs. (j, l) 36 hours after siRNA transfection, fibers (j) or SCs (l) were harvested for EdU detection and immunostaining. (k, m) Quantification of EdU^{+} SCs after siRNA transfection. In (k), n=3 independent experiments, the number of quantified fibers is 72 and 75 for siNC and siCPEB1 respectively. In (m), n=3 independent experiments.

(n, o) Analysis of $Ki67^{+}$ proliferating SCs after CPEB1 knockdown. (n) 36 hours after siRNA transfection, SCs were fixed, followed by Ki67 immunostaining. (o) Quantification of the number of $Ki67^{+}$ SCs after siRNA transfection. (n=3 independent experiments).

Data are presented as mean ± SD in b, f, h, k, m, and o. The *p* values calculated by two-tailed unpaired t test were used for comparing two groups in b, f, h, k, m and o, ns not significant. Source data is provided as a Source Data file.

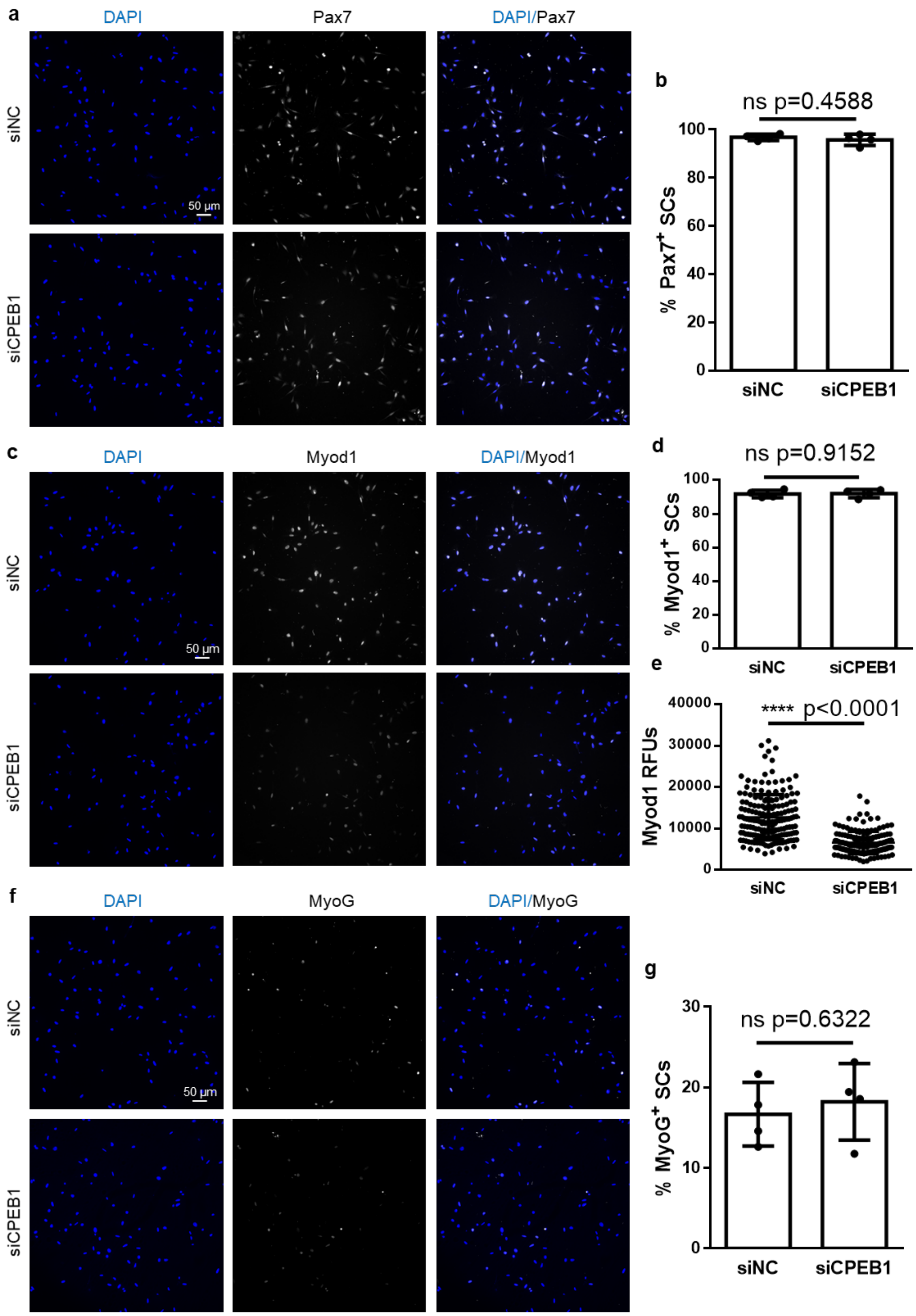
a
DAPI
Pax7
DAPI/Pax7
siNC
siCPEB1
50 µm
b
ns p=0.4588
% Pax7+ SCs
100
80
60
40
20
0
siNC
siCPEB1
c
DAPI
Myod1
DAPI/Myod1
siNC
siCPEB1
50 µm
d
ns p=0.9152
% Myod1+ SCs
100
80
60
40
20
0
siNC
siCPEB1
e
**** p<0.0001
Myod1 RFUs
40000
30000
20000
10000
0
siNC
siCPEB1
f
DAPI
MyoG
DAPI/MyoG
siNC
siCPEB1
50 µm
g
ns p=0.6322
% MyoG+ SCs
30
20
10
0
siNC
siCPEB1

Supplementary Fig. 4 (associated to Fig. 2). CPEB1 knockdown did not affect the percentages of Pax7, Myod1, MyoG positive SCs during activation

(a, b) Percentage of $Pax7^+$ SCs after CPEB1 knockdown. (a) 48 hours after siRNA transfection, SCs were harvested for immunostaining of Pax7. Nuclei were stained with DAPI. (b) Percentage of $DAPI^+$ SCs which are $Pax7^+$ after siRNA transfection. (n=4 independent experiments).

(c - e) Percentage of $Myod1^+$ SCs after CPEB1 knockdown. (c) 48 hours after siRNA transfection, SCs were harvested for immunostaining of Myod1. Nuclei were stained with DAPI. (d) Percentage of $DAPI^+$ SCs which were $Myod1^+$ after siRNA transfection. (n=4 independent experiments). (e) Quantification of Myod1 RFUs (the number of quantified cells is 180 and 159 for siNC and siCPEB1 respectively).

(f, g) Percentage of $MyoG^+$ SCs after CPEB1 knockdown. (f) 48 hours after siRNA transfection, SCs were harvested for immunostaining of MyoG. Nuclei were stained with DAPI. (g) Percentage of $DAPI^+$ SCs that were $MyoG^+$ after siRNA transfection. (n=4 independent experiments).

Data are presented as mean ± SD in b, d, e and g. The *p* values calculated by two-tailed unpaired t test were used for comparing two groups in b, d, e, g, ns not significant. Source data is provided as a Source Data file.

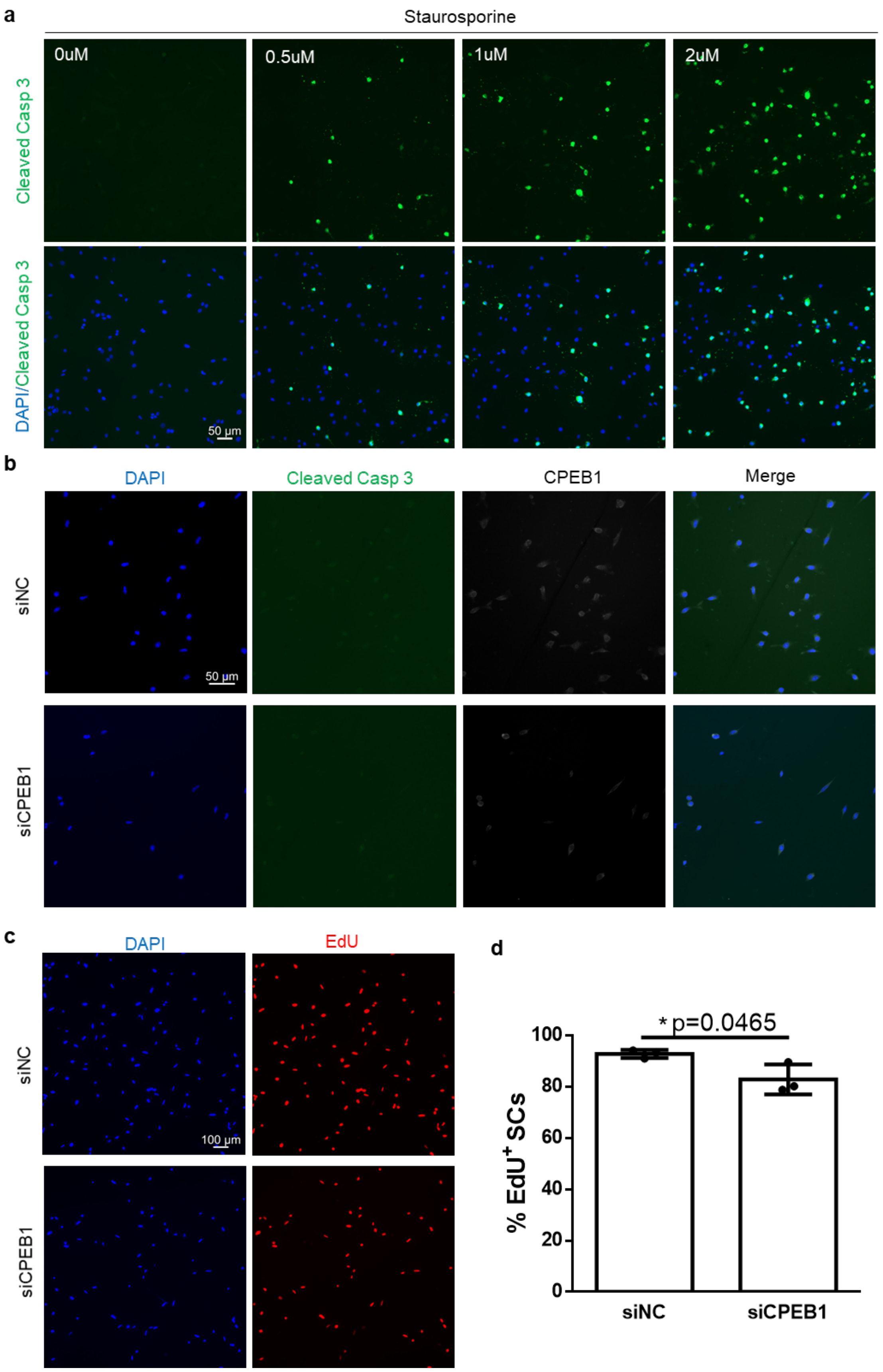
a
Staurosporine
0uM
0.5uM
1uM
2uM
Cleaved Casp 3
DAPI/Cleaved Casp 3
50 µm
b
DAPI
Cleaved Casp 3
CPEB1
Merge
siNC
siCPEB1
50 µm
c
DAPI
EdU
siNC
siCPEB1
100 µm
d
* p=0.0465
% EdU+ SCs
100
80
60
40
20
0
siNC
siCPEB1

Supplementary Fig. 5 (associated to Fig. 2). CPEB1 knockdown did not induce SC apoptosis

(a) Cleaved Caspase 3 antibody specificity test performed on C2 cells. C2 cells were treated with increasing concentrations of the apoptosis inducer, Staurosporine for 6 hours followed by cleaved Caspase 3 immunostaining. Nuclei were stained with DAPI. (n=3 independent experiments).

(b) Cleaved Caspase 3 immunostaining performed on SCs 48 hours after CPEB1 knockdown. (n=3 independent experiments).

(c, d) EdU incorporation analysis in SCs after CPEB1 knockdown. (c) 72 hours after siRNA transfection, SCs were harvested for EdU detection. Nuclei were stained with DAPI. (d) Quantification of EdU^+ SCs after siRNA transfection. (n=3 independent experiments).

Data are presented as mean ± SD in d. The *p* values calculated by two-tailed unpaired t test were used for comparing two groups in d. Source data is provided as a Source Data file.

a

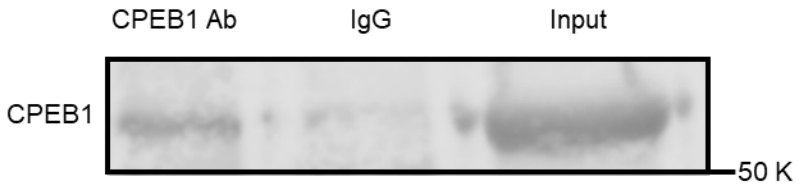

Supplementary Fig. 6 (associated to Fig. 3). CPEB1 antibody immunoprecipitation followed by western blot

(a) CPEB1 western blot after CPEB1 antibody immunoprecipitation. 48 hours after plating down, the SCs were harvested for CPEB1 immunoprecipitation followed by western blot detection of CPEB1 protein. (n=3 independent experiments). Source data is provided as a Source Data file.

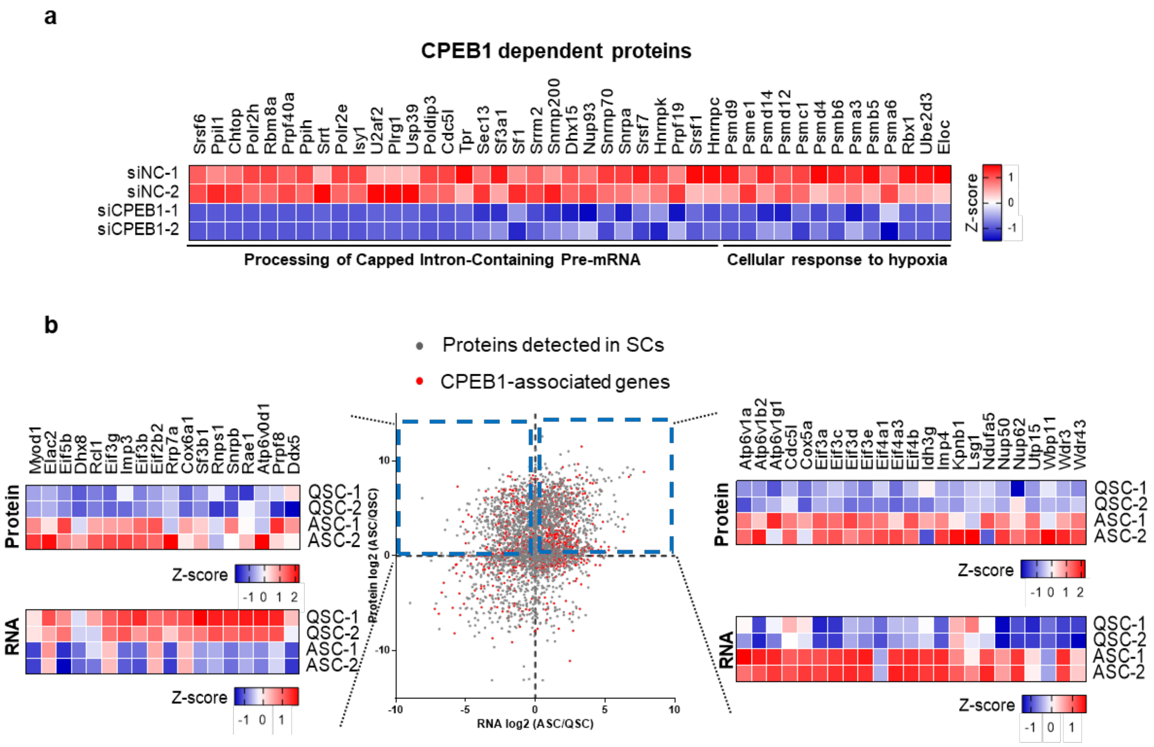

Supplementary Fig. 7 (associated to Fig. 4). CPEB1 regulates the translational landscape during SC activation

(a) Heatmap of representative protein expression for processing of capped intron-containing pre-mRNA and cellular response to hypoxia pathways. (n=2 independent experiments).

(b) Scatter plot of the fold change of RNA and protein expression by comparing ASCs to QSCs. The red dots show the CPEB1-associated genes. Heatmap shows the representative CPEB1-associated genes with upregulated protein expression and downregulated transcripts (left) or upregulated transcripts (right) during SC activation.

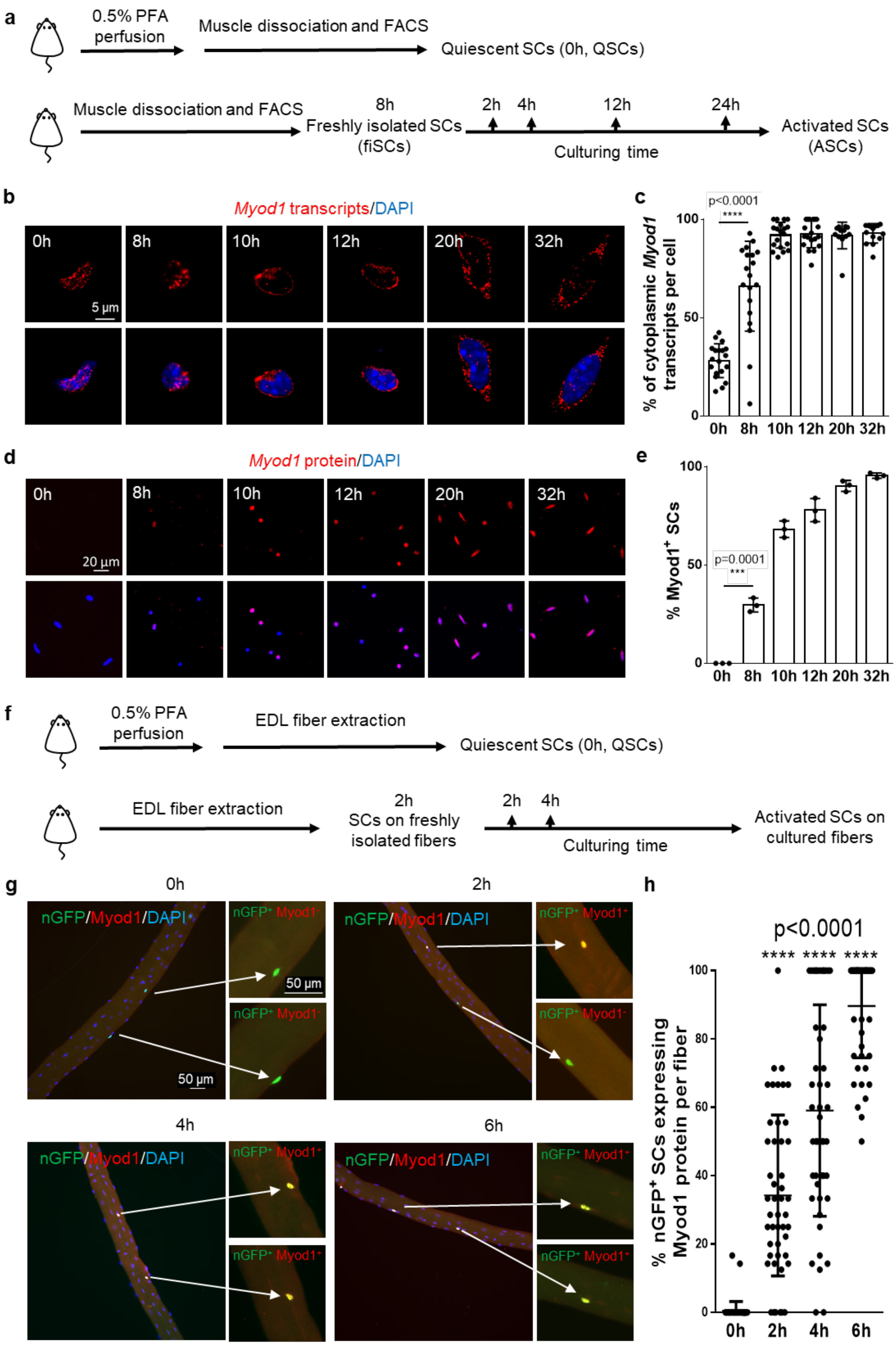
a
0.5% PFA perfusion
Muscle dissociation and FACS
Quiescent SCs (0h, QSCs)
Muscle dissociation and FACS
8h
Freshly isolated SCs (fiSCs)
2h
4h
12h
24h
Culturing time
Activated SCs (ASCs)
b
Myod1 transcripts/DAPI
0h
8h
10h
12h
20h
32h
5 µm
c
p<0.0001
****
% of cytoplasmic Myod1 transcripts per cell
100
50
0
0h
8h
10h
12h
20h
32h
d
Myod1 protein/DAPI
0h
8h
10h
12h
20h
32h
20 µm
e
% Myod1+ SCs
p=0.0001
***
100
50
0
0h
8h
10h
12h
20h
32h
f
0.5% PFA perfusion
EDL fiber extraction
Quiescent SCs (0h, QSCs)
EDL fiber extraction
2h
SCs on freshly isolated fibers
2h
4h
Culturing time
Activated SCs on cultured fibers
g
0h
2h
4h
6h
nGFP/Myod1/DAPI
nGFP+ Myod1-
nGFP+ Myod1+
50 µm
h
p<0.0001
****
% nGFP+ SCs expressing Myod1 protein per fiber
100
80
60
40
20
0
0h
2h
4h
6h

Supplementary Fig. 8 (associated to Fig. 5). ***Myod1*** **transcripts undergo transportation to the cytoplasm for its rapid protein expression upon SC dissociation from the niche**

(a - e) *Myod1* transcripts, protein expression and subcellular localization analysis during SC activation. (a) Timeline of isolation of quiescent SCs (QSCs, isolated from perfused mice) and SCs activated *in vitro* for different durations for (b - e). (b) Single-molecule fluorescence *in situ* hybridization (smFISH) of *Myod1* transcripts in QSCs and ASCs. Representative images of *Myod1* transcripts smFISH are shown. Nuclei were stained with DAPI. (c) Quantification of the percentage of *Myod1* transcripts with different subcellular localizations in SCs. (n=3 independent experiments, the number of quantified SCs at 0 hours (h), 8h,10h, 12h, 20h and 32h are 19, 19, 19, 19, 12 and, 13 respectively). (d) Myod1 protein was immunostained on QSCs and ASCs. Representative images of Myod1 immunostaining are shown. Nuclei were stained with DAPI. (e) Quantification of the percentage of $Myod1^{+}$ SCs. (n=3, independent experiments).

(f - h) Myod1 protein expression analysis on SCs per myofiber during activation. (f) Timeline showing isolation of EDL muscle fibers and *in vitro* culture for SC activation. (g) Representative immunostaining images of Myod1 and GFP on fibers are shown. (h) Quantification of the percentage of $Myod1^{+}$ SCs during activation. (n=3 independent experiments, the number of quantified SCs at 0h, 2h, 4h and 6h are 64, 47, 47 and 47 respectively).

Data are presented as mean ± SD in c, e, and h. The *p* values calculated by two-tailed unpaired t test were used for comparing two groups in c, e, and h. Source data is provided as a Source Data file.

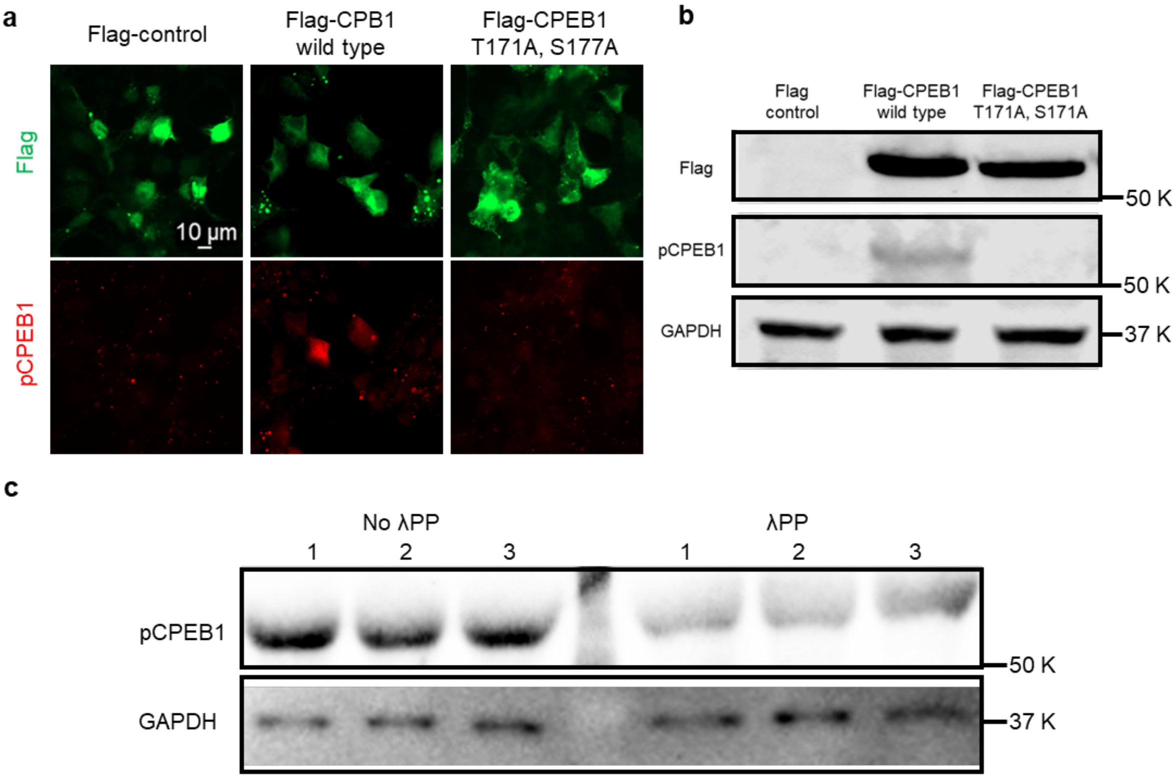

d

ELISA (OD450)									
Antibody dilution	1250	2500	5000	10000	20000	40000	80000	Rabbit IgG (1:1250)	
pCPEB1 antibody	2.808	2.748	2.436	2.216	1.652	1.2495	0.9905	0.146	phosphor-peptides
pCPEB1 antibody	0.132	0.1325	0.1255	0.139	0.141	0.1355	0.1445	0.1435	non-phosphor-peptides

Supplementary Fig. 9 (associated to Fig. 6). pCPEB1 antibody specificity test

(a, b) Phosphor-CPEB1 (pCPEB1) antibody specificity test performed on 293T cells after overexpression of WT CPEB1 or its mutant form. Immunostaining (a) (n=3 independent experiments) and western blot (b) (n=3 independent experiments) of pCPEB1 and Flag-tagged proteins after transfection of plasmids carrying Flag-tagged WT CPEB1 or its mutant into 293T cells. GAPDH used as a loading control.

(c) Primary myoblasts harvested in RIPA buffer were treated with or without λ protein phosphatase (λPP). Afterwards, a western blot for pCPEB1 was performed. GAPDH was used as internal control. (n=3 independent experiments).

(d) OD450 measurements of ELISA using pCPEB1 antibody to detect either phosphor-peptides (GSRLD(p-T) RPILD (p-S) RSSS) or non-phosphor-peptides (GSRLDTRPILDSRSSS). (n=2 independent experiments). Rabbit IgG antibody was used as negative control. Source data is provided as a Source Data file.

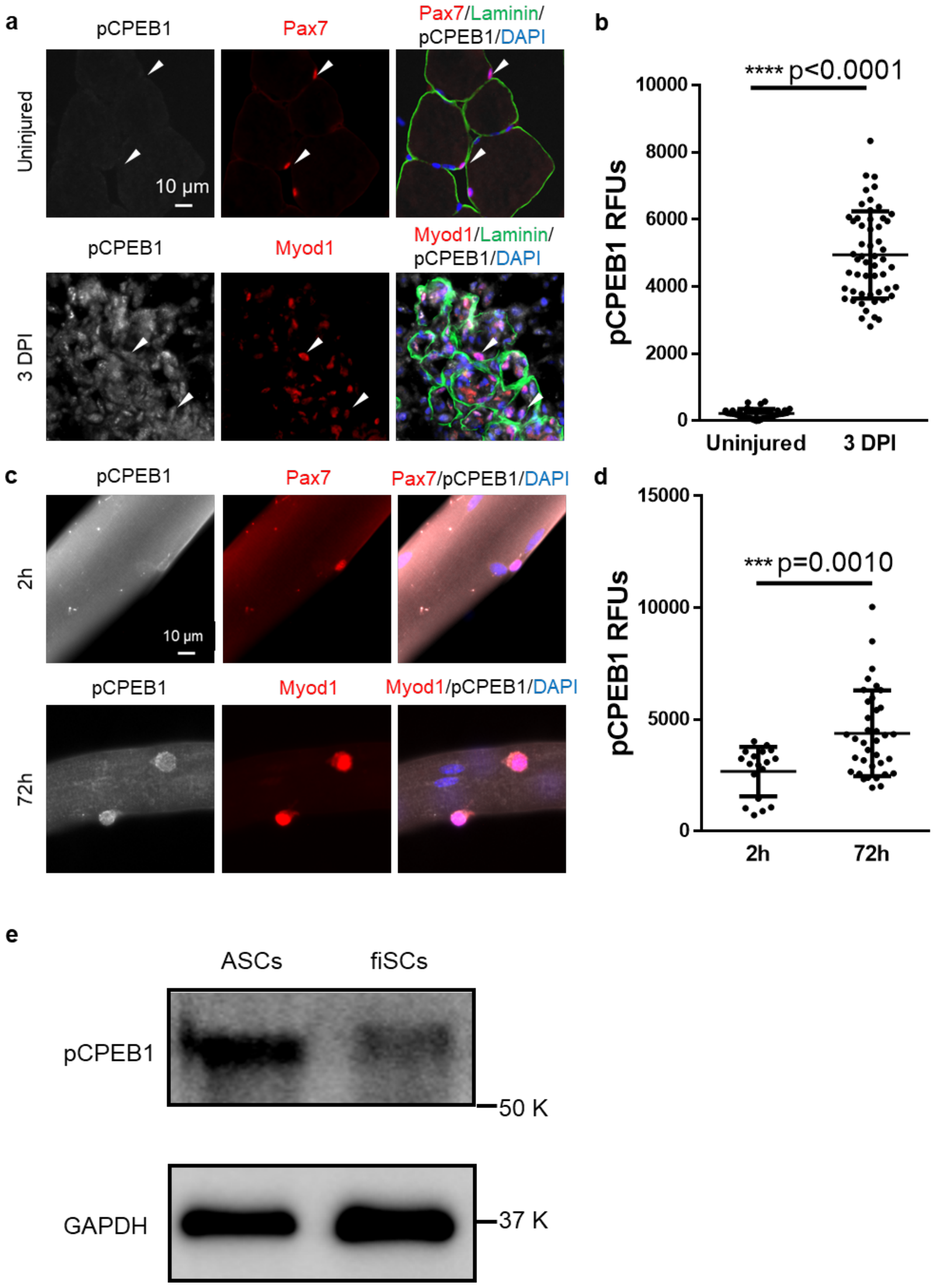

a
pCPEB1
Pax7
Pax7/Laminin/
pCPEB1/DAPI
Uninjured
10 µm
pCPEB1
Myod1
Myod1/Laminin/
pCPEB1/DAPI
3 DPI
b
**** p<0.0001
pCPEB1 RFUs
10000
8000
6000
4000
2000
0
Uninjured
3 DPI
c
pCPEB1
Pax7
Pax7/pCPEB1/DAPI
2h
10 µm
pCPEB1
Myod1
Myod1/pCPEB1/DAPI
72h
d
15000
*** p=0.0010
10000
5000
0
pCPEB1 RFUs
2h
72h
e
ASCs
fiSCs
pCPEB1
50 K
GAPDH
37 K

Supplementary Fig. 10 (associated to Fig. 6). CPEB1 is phosphorylated during SC activation

(a, b) Phosphor-CPEB1 expression analysis of SCs on TA muscle sections after injury. (a) Uninjured and 3 days post injured (DPI) TA muscles were cryo-sectioned, followed by pCPEB1 immunostaining on QSCs ($Pax7^+$) or ASCs ($Myod1^+$). Nuclei were stained with DAPI. (b) Quantification of pCPEB1 RFUs in SCs after muscle injury. (n=3 independent experiments, the number of quantified SCs in uninjured muscle is 44, for 3 DPI muscle, 54).

(c, d) Phosphor-CPEB1 expression analysis of fiber-associated SCs during activation. (c) Immunostaining of pCPEB1 on freshly extracted (2h) and cultured fibers (72h). (d) Quantification of pCPEB1 protein levels on SCs. (n=3 independent experiments, the number of quantified SCs at 2h is 18, and at 72h is 36).

(e) Western blot of pCPEB1 on fiSCs and ASCs (SCs sorted from 3 days post-injured muscles). GAPDH protein used as internal control. (n=3 independent experiments).

Data are presented as mean ± SD in b and d. The *p* values calculated by two-tailed unpaired t test were used for comparing two groups in b and d. Source data is provided as a Source Data file.

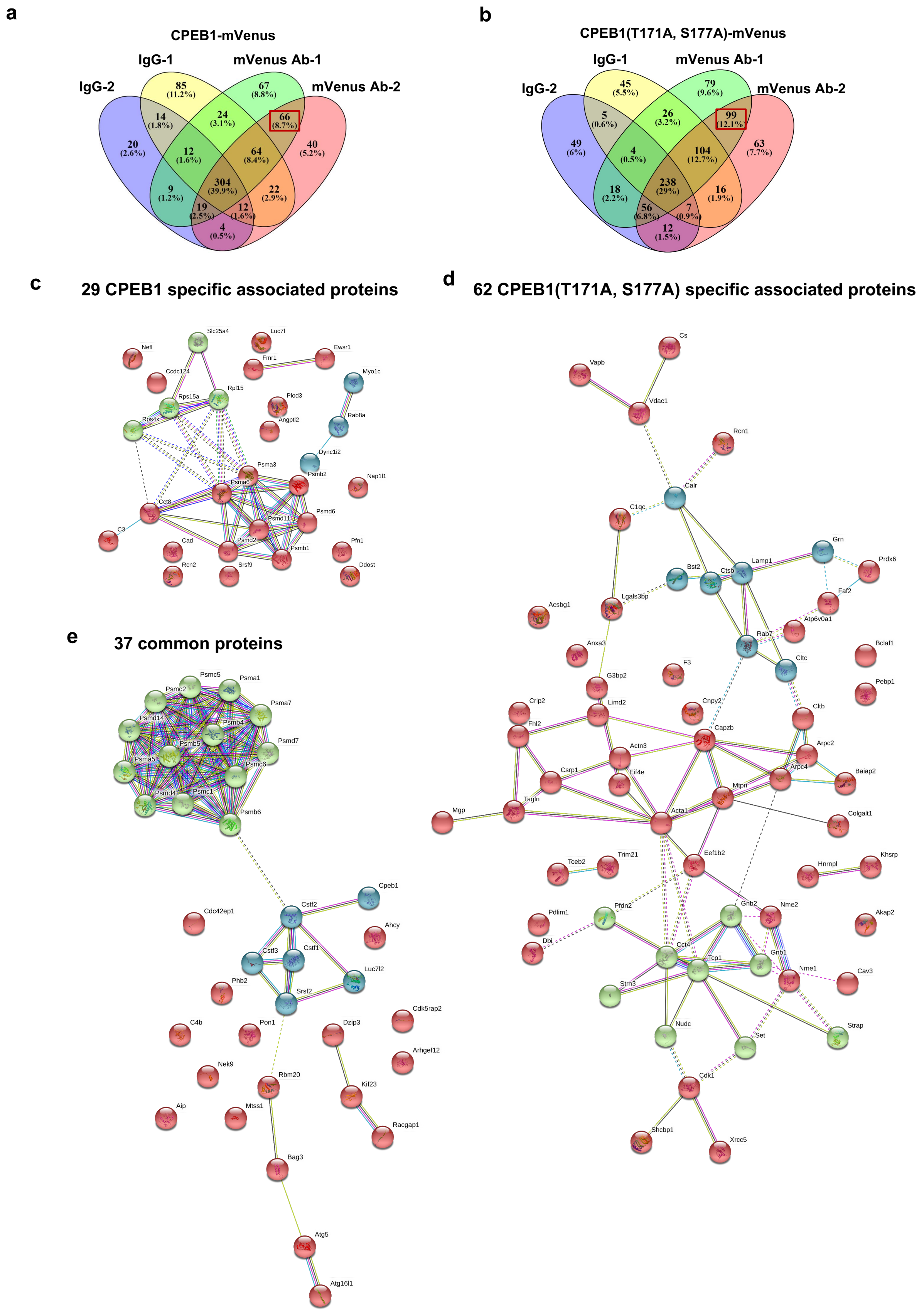

a
CPEB1-mVenus
IgG-1
mVenus Ab-1
IgG-2
mVenus Ab-2
85 (11.2%)
67 (8.8%)
14 (1.8%)
24 (3.1%)
66 (8.7%)
20 (2.6%)
12 (1.6%)
64 (8.4%)
40 (5.2%)
304 (39.9%)
9 (1.2%)
22 (2.9%)
19 (2.5%)
12 (1.6%)
4 (0.5%)
b
CPEB1(T171A, S177A)-mVenus
IgG-1
mVenus Ab-1
IgG-2
mVenus Ab-2
45 (5.5%)
79 (9.6%)
5 (0.6%)
26 (3.2%)
99 (12.1%)
49 (6%)
4 (0.5%)
104 (12.7%)
63 (7.7%)
238 (29%)
18 (2.2%)
16 (1.9%)
56 (6.8%)
7 (0.9%)
12 (1.5%)
c
29 CPEB1 specific associated proteins
d
62 CPEB1(T171A, S177A) specific associated proteins
e
37 common proteins

Supplementary Fig. 11 (associated to Fig. 6). Analysis of the CPEB1 interacting protein network

(a, b) Identification of proteins interacting with CPEB1 or CPEB1 (T171A, S177A). Venn diagram of proteins detected by mass spectrometry after IgG or mVenus antibody immunoprecipitation in C2 cells transfected with (a) CPEB1-mVenus or (b) CPEB1 (T171A, S177A)-mVenus. The red rectangle shows the proteins targeted by CPEB1 or mutated CPEB1.

(c - e) The functional protein association networks of (c) 29 CPEB1-specific associated proteins, (d) 62 CPEB1 (T171, S177A)-specific associated proteins, or (e) 37 common proteins that interacted with wild type CPEB1 and T171A, S177A mutated CPEB1.

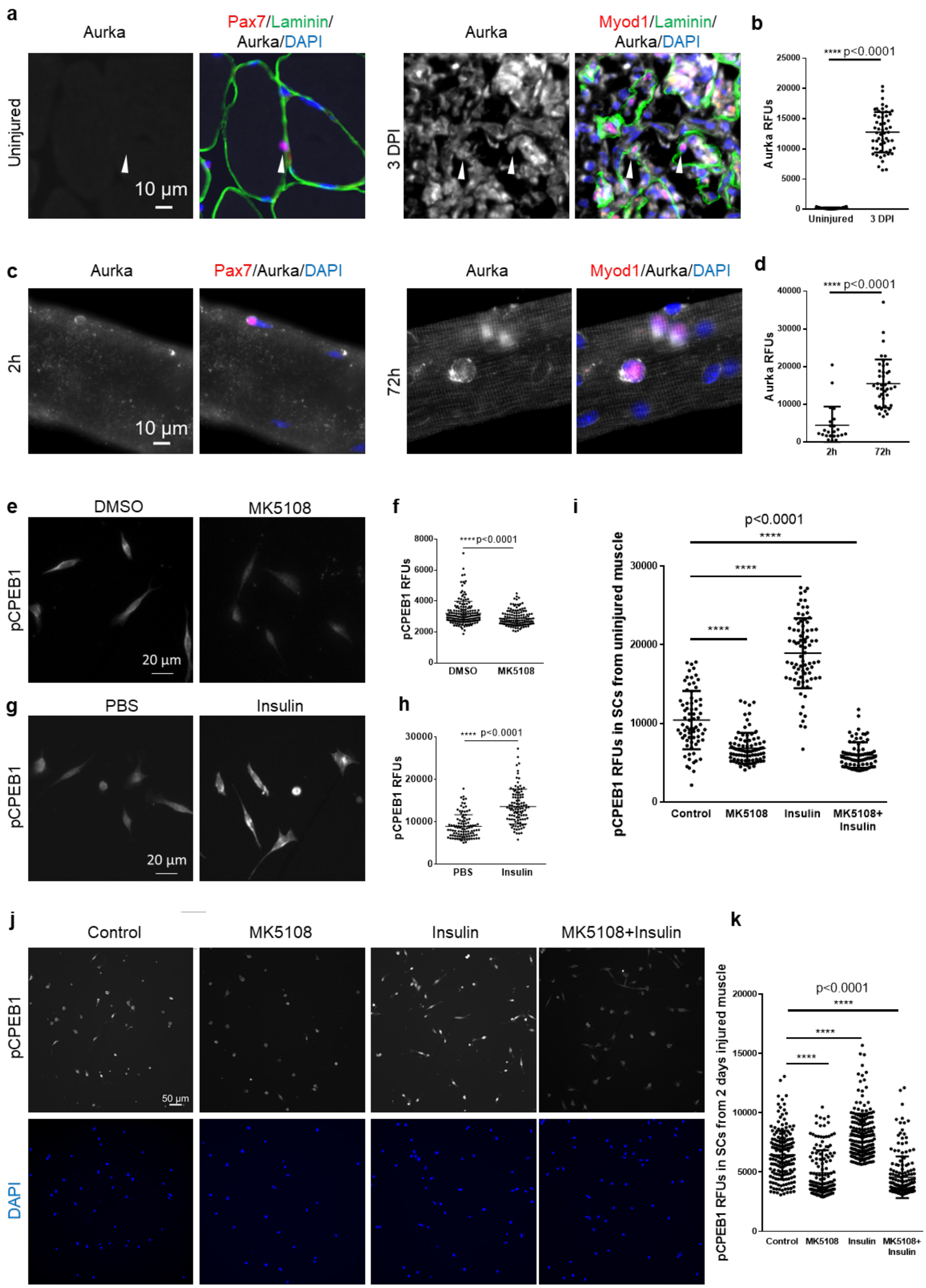

a
Aurka
Pax7/Laminin/Aurka/DAPI
Aurka
Myod1/Laminin/Aurka/DAPI
Uninjured
3 DPI
10 µm
b
**** p<0.0001
Aurka RFUs
Uninjured
3 DPI
c
Aurka
Pax7/Aurka/DAPI
Aurka
Myod1/Aurka/DAPI
2h
72h
10 µm
d
**** p<0.0001
Aurka RFUs
2h
72h
e
DMSO
MK5108
pCPEB1
20 µm
f
****p<0.0001
pCPEB1 RFUs
DMSO
MK5108
g
PBS
Insulin
pCPEB1
20 µm
h
**** p<0.0001
pCPEB1 RFUs
PBS
Insulin
i
p<0.0001
pCPEB1 RFUs in SCs from uninjured muscle
Control
MK5108
Insulin
MK5108+ Insulin
j
Control
MK5108
Insulin
MK5108+Insulin
pCPEB1
DAPI
50 µm
k
p<0.0001
pCPEB1 RFUs in SCs from 2 days injured muscle
Control
MK5108
Insulin
MK5108+ Insulin

Supplementary Fig. 12 (associated to Fig. 7). Aurka is upregulated during SC activation and regulates CPEB1 phosphorylation

(a - d) Analysis of Aurora kinase A (Aurka) protein expression in SCs. (a) Uninjured and 3 DPI TA muscles were cryo-sectioned, followed by Aurka immunostaining on $Pax7^+$ or $Myod1^+$ SCs. Nuclei were stained with DAPI. (b) Quantification of Aurka protein levels in SCs after muscle injury. (n=3 independent experiments, the number of quantified SCs in uninjured muscle is 36, for 3 DPI muscle, 55). (c) Aurka immunostaining on freshly extracted (2h) and cultured fibers (72h). (d) Quantification of Aurka protein levels in SCs. (n=3 independent experiments, the number of quantified SCs in 2h fibers is 24, for 72h fibers, 41).

(e - h) Analysis of Phosphor-CPEB1 expression after Aurka inhibitor (MK5108) or activator (insulin) treatment. (e) After FACS-isolation, SCs were treated with MK5108 for 36 hours followed by immunostaining of pCPEB1 on SCs. (f) Quantification of pCPEB1 RFUs after MK5108 treatment. (n=3 independent experiments, the number of quantified SCs in control and MK5108 groups are 171 and 134 respectively). (g) After FACS-isolation, SCs were treated with insulin for 36 hours followed by pCPEB1 immunostaining on SCs. (h) Quantification of pCPEB1 RFUs after insulin treatment. (n=3 independent experiments, the number of quantified SCs in control and insulin groups are 103 and 106 respectively).

(i) Quantification of pCPEB1 RFUs in SCs as shown in Fig. 7a. (n=3 independent experiments. The number of quantified SCs of control, MK5108, Insulin and MK5108+Insulin groups are 72, 86, 81 and 78 respectively).

(j, k) TA muscles injured with $BaCl_2$ were injected with insulin and/or MK5108 two times following the injection scheme as shown in Fig. 7c. (j) 2 days after injury, TA muscles were harvested for isolation of ASCs, followed by pCPEB1 immunostaining. Nuclei were stained by DAPI. (k) Quantification of pCPEB1 RFUs in SCs. (n=3 independent experiments, the number of quantified SCs of control, MK5108, Insulin and MK5108+Insulin groups are 170, 144, 211 and 175 respectively).

Data are presented as mean ± SD in b, d, f, h, i and k. The *p* values calculated by two-tailed unpaired t test were used for comparing two groups in b, d, f, h, i, k, ns not significant. Source data is provided as a Source Data file.

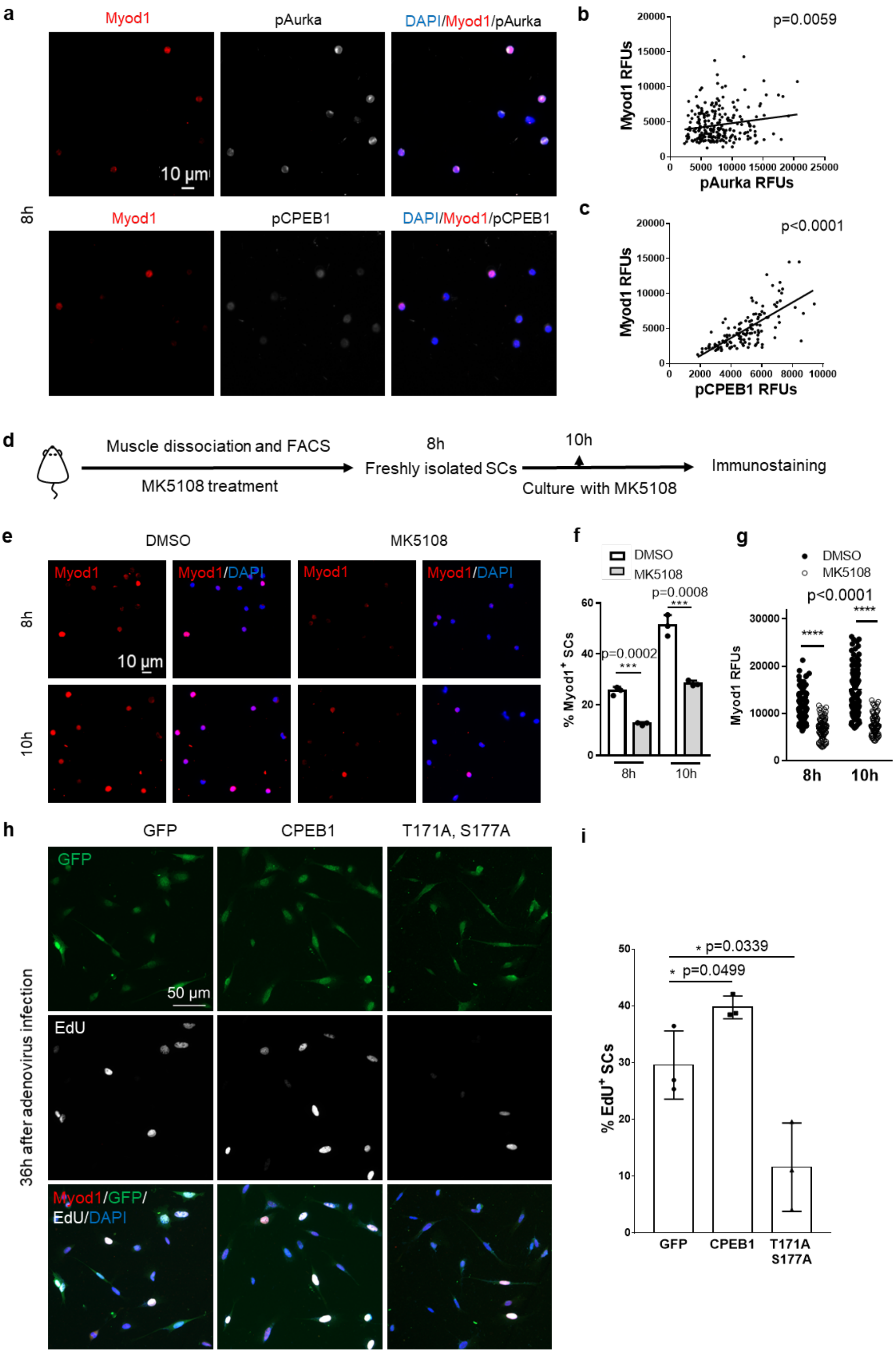
a
Myod1
pAurka
DAPI/Myod1/pAurka
10 μm
8h
Myod1
pCPEB1
DAPI/Myod1/pCPEB1
b
p=0.0059
Myod1 RFUs
pAurka RFUs
c
p<0.0001
Myod1 RFUs
pCPEB1 RFUs
d
Muscle dissociation and FACS
MK5108 treatment
8h
Freshly isolated SCs
10h
Culture with MK5108
Immunostaining
e
DMSO
MK5108
Myod1
Myod1/DAPI
8h
10h
10 μm
f
DMSO
MK5108
p=0.0008
p=0.0002
% Myod1+ SCs
8h
10h
g
DMSO
MK5108
p<0.0001
Myod1 RFUs
8h
10h
h
GFP
CPEB1
T171A, S177A
36h after adenovirus infection
50 μm
EdU
Myod1/GFP/
EdU/DAPI
i
p=0.0339
p=0.0499
% EdU+ SCs
GFP
CPEB1
T171A
S177A

Supplementary Fig. 13 (associated to Fig. 7). CPEB1 phosphorylation is required for SC activation

(a - c) Correlation of Myod1 protein between pAurka or pCPEB1 in fiSCs. (a) After FACS-isolation, SCs were subjected to immunostaining for Myod1 and pAurka or pCPEB1. (b, c) Pearson correlation analysis between Myod1 protein RFUs and (b) pAurka RFUs or (c) pCPEB1 RFUs. (n=3 independent experiments, the number of quantified cells in (b) is 239, and in (c) is 128).

(d - g) Myod1 protein expression analysis in SCs after inhibiting CPEB1 phosphorylation. (d) Timeline of the MK5108 treatment for (e - g). (e) Hindlimb muscles were treated with MK5108 during FACS sample preparation and *in vitro* culture. SCs were subsequently harvested for Myod1 immunostaining. Nuclei were stained with DAPI. (f, g) Quantification of the percentage of (f) Myod1^{+} SCs and (g) Myod1 protein RFUs in SCs after MK5108 treatment. (n=3 independent experiments, the number of quantified SCs in DMSO and MK5108 treatment groups at 8h are 107 and 119; at 10h are 114 and 84 respectively).

(h, i) EdU incorporation analysis on SCs after wild type or phosphor-mutant CPEB1 overexpression. (h) After FACS-isolation, SCs were infected with adenovirus expressing GFP, wild-type CPEB1, or mutant CPEB1 (T171A, S177A). Throughout the experiment, SCs were cultured with EdU. After culturing for 36 hours, SCs were harvested for EdU detection and immunostaining for Myod1. Nuclei were stained with DAPI. (i) Quantification of the percentage of EdU^{+} SCs after indicated virus infection. (n=3 independent experiments).

In b and c, Pearson correlation analysis was performed. Data are presented as mean ± SD in f, g and i. The *p* values calculated by two-tailed unpaired t test were used for comparing two groups in f, g and i. Source data is provided as a Source Data file.

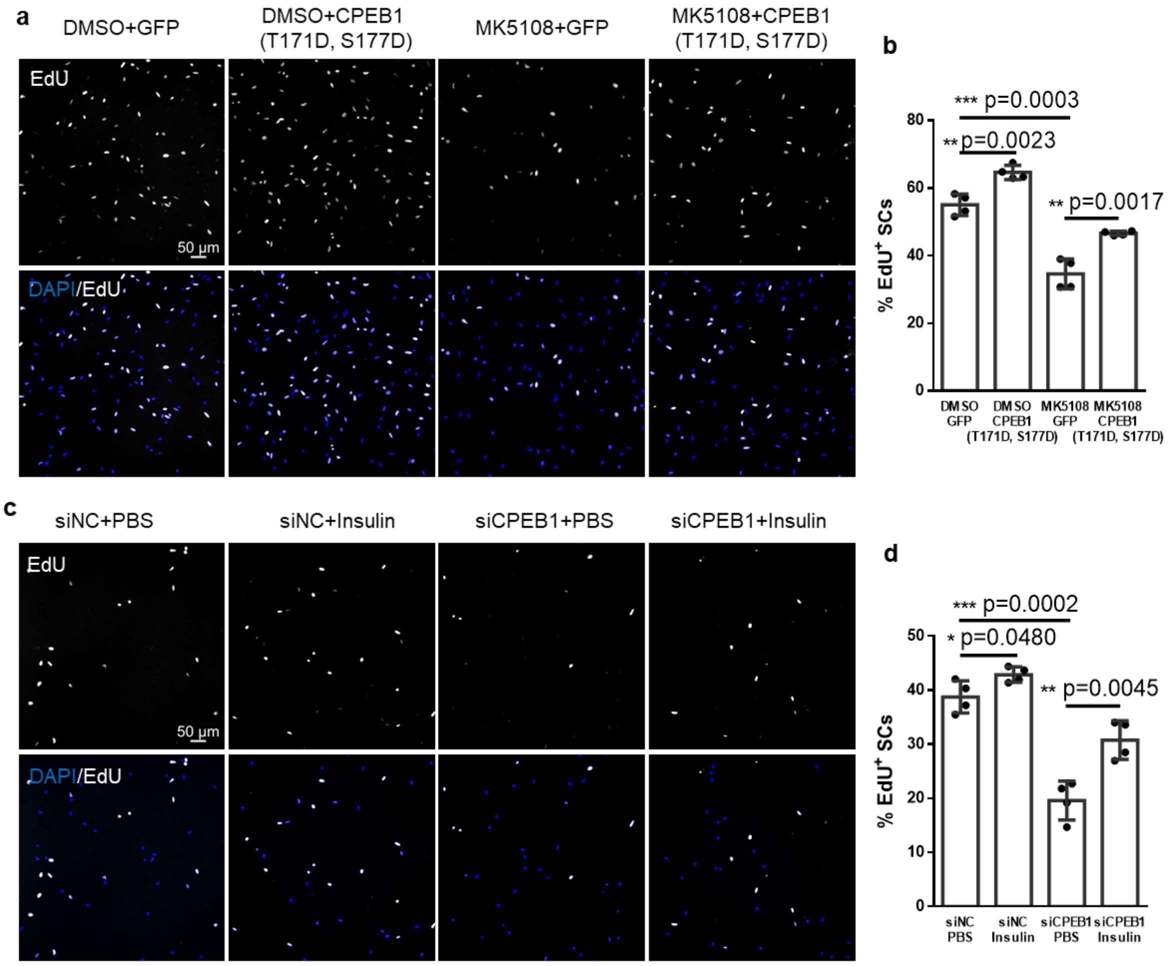
a
DMSO+GFP
DMSO+CPEB1 (T171D, S177D)
MK5108+GFP
MK5108+CPEB1 (T171D, S177D)
EdU
DAPI/EdU
50 μm
b
*** p=0.0003
** p=0.0023
** p=0.0017
% EdU+ SCs
80
60
40
20
0
DMSO GFP
DMSO CPEB1 (T171D, S177D)
MK5108 GFP
MK5108 CPEB1 (T171D, S177D)
c
siNC+PBS
siNC+Insulin
siCPEB1+PBS
siCPEB1+Insulin
EdU
DAPI/EdU
50 μm
d
*** p=0.0002
* p=0.0480
** p=0.0045
% EdU+ SCs
50
40
30
20
10
0
siNC PBS
siNC Insulin
siCPEB1 PBS
siCPEB1 Insulin

Supplementary Fig. 14 (associated to Fig. 7). MK5108 or insulin regulates SC activation partially through the CPEB1 pathway

(a, b) Analysis of EdU incorporation in SCs after CPEB1 (T171D, S177D) overexpression and MK5108 treatment. (a) fiSCs treated with DMSO or MK5108 were infected with adenovirus carrying GFP or CPEB1 (T171D, S177D). Meanwhile, EdU was added to the culture medium. SCs were harvested for EdU detection 36 hours after treatment. (b) Quantification of the percentage of EdU^{+} SCs after indicated treatment. (n=4 independent experiments).

(c, d) Analysis of EdU incorporation in SCs after siCPEB1 transfection and insulin treatment. (c) fiSCs were transfected with siRNA targeting CPEB1 with the addition of insulin. Meanwhile, EdU was added to the culture medium. SCs were harvested for EdU detection 36 hours after treatment. (d) Quantification of the percentage of EdU^{+} SCs after indicated treatment. (n=4 independent experiments).

Data are presented as mean ± SD in b and d. The *p* values calculated by two-tailed unpaired t test were used for comparing two groups in b and d. Source data is provided as a Source Data file.

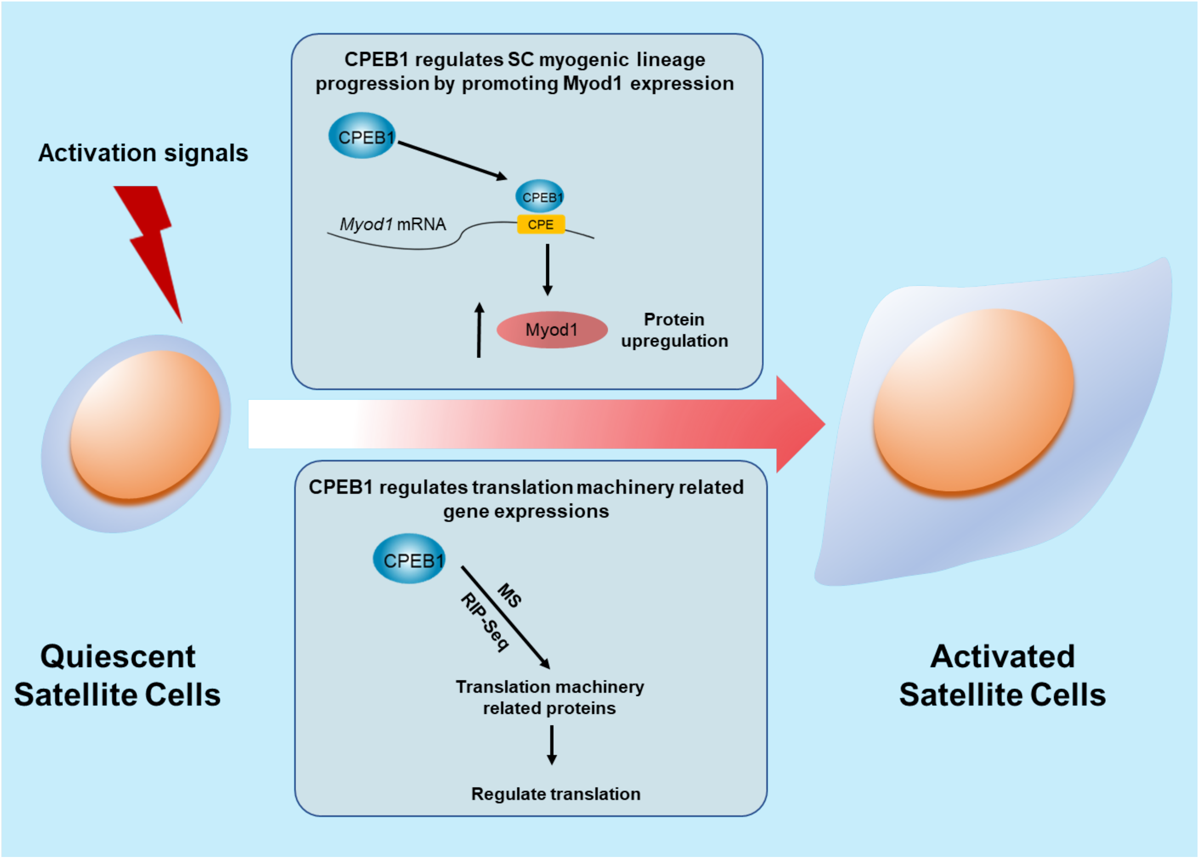

Supplementary Fig. 15. Working model: CPEB1-mediated translation reprogramming directs SC activation
