# Supplementary material for: CPEB1 directs muscle stem cell activation by reprogramming the translational landscape
Source: Nat Commun. 2022 Feb 17;13:947. doi: 10.1038/s41467-022-28612-1 (PMC8854658; doi:10.1038/s41467-022-28612-1)
Supplement: Supplementary file 3 — Description of Additional Supplementary Files [file 41467_2022_28612_MOESM3_ESM.docx]

**Description of Additional Supplementary Files**

File name: Supplementary Data 1

Description: Proteome analysis of QSC, fiSC, cASC, iASC.

File name: Supplementary Data 2

Description: Gene fold change of proteome and transcriptome.

File name: Supplementary Data 3

Description: CPEB1 RIP-Seq analysis.

File name: Supplementary Data 4

Description: siNC and siCPEB1 proteome analysis.

File name: Supplementary Data 5

Description: CPEB1 or CPEB1 (T171A, S177A) interacting proteome analysis.
